# R5 Clade C SHIV Strains with Tier 1 or 2 Neutralization Sensitivity: Tools to Dissect Env Evolution and to Develop AIDS Vaccines in Primate Models

**DOI:** 10.1371/journal.pone.0011689

**Published:** 2010-07-21

**Authors:** Nagadenahalli B. Siddappa, Jennifer D. Watkins, Klemens J. Wassermann, Ruijiang Song, Wendy Wang, Victor G. Kramer, Samir Lakhashe, Michael Santosuosso, Mark C. Poznansky, Francis J. Novembre, François Villinger, James G. Else, David C. Montefiori, Robert A. Rasmussen, Ruth M. Ruprecht

**Affiliations:** 1 Dana-Farber Cancer Institute, Boston, Massachusetts, United States of America; 2 Harvard Medical School, Boston, Massachusetts, United States of America; 3 Partners AIDS Research Center and Infectious Diseases Medicine, Massachusetts General Hospital (East), Charlestown, Massachusetts, United States of America; 4 Yerkes National Primate Research Center, Emory University, Atlanta, Georgia, United States of America; 5 Department of Microbiology and Immunology, Emory University, Atlanta, Georgia, United States of America; 6 Division of Pathology and Laboratory Medicine, Emory University, Atlanta, Georgia, United States of America; 7 Department of Surgery, Duke University Medical Center, Durham, North Carolina, United States of America; Institut Pasteur Korea, Republic of Korea

## Abstract

**Background:**

HIV-1 clade C (HIV-C) predominates worldwide, and anti-HIV-C vaccines are urgently needed. Neutralizing antibody (nAb) responses are considered important but have proved difficult to elicit. Although some current immunogens elicit antibodies that neutralize highly neutralization-sensitive (tier 1) HIV strains, most circulating HIVs exhibiting a less sensitive (tier 2) phenotype are not neutralized. Thus, both tier 1 and 2 viruses are needed for vaccine discovery in nonhuman primate models.

**Methodology/Principal Findings:**

We constructed a tier 1 simian-human immunodeficiency virus, SHIV-1157ipEL, by inserting an “early,” recently transmitted HIV-C *env* into the SHIV-1157ipd3N4 backbone [1] encoding a “late” form of the same *env,* which had evolved in a SHIV-infected rhesus monkey (RM) with AIDS. SHIV-1157ipEL was rapidly passaged to yield SHIV-1157ipEL-p, which remained exclusively R5-tropic and had a tier 1 phenotype, in contrast to “late” SHIV-1157ipd3N4 (tier 2). After 5 weekly low-dose intrarectal exposures, SHIV-1157ipEL-p systemically infected 16 out of 17 RM with high peak viral RNA loads and depleted gut CD4^+^ T cells. SHIV-1157ipEL-p and SHIV-1157ipd3N4 *env* genes diverge mostly in V1/V2. Molecular modeling revealed a possible mechanism for the increased neutralization resistance of SHIV-1157ipd3N4 Env: V2 loops hindering access to the CD4 binding site, shown experimentally with nAb b12. Similar mutations have been linked to decreased neutralization sensitivity in HIV-C strains isolated from humans over time, indicating parallel HIV-C Env evolution in humans and RM.

**Conclusions/Significance:**

SHIV-1157ipEL-p, the first tier 1 R5 clade C SHIV, and SHIV-1157ipd3N4, its tier 2 counterpart, represent biologically relevant tools for anti-HIV-C vaccine development in primates.

## Introduction

Recent developments in AIDS vaccine research focused attention on the need for developing vaccine strategies that can generate both cellular and humoral immunity [2]. Currently, T-cell as well as nAb-based responses are believed to be necessary for eliciting an effective response against HIV-1 (reviewed in [3]). During the course of natural HIV-1 infection, nAbs are generated that may delay disease progression (reviewed in [4]).

More than 90% of all HIV-1 transmissions occur mucosally, and almost all of these infections are initiated by R5 strains – even when the source persons have mixed infections [5]. Therefore, preclinical primate model studies should focus on vaccine prevention of mucosal R5 virus transmission. The availability of a primate model that reflects the salient biologic features of HIV-1 transmission among humans will enhance our understanding of lentiviral pathogenesis and facilitate the development of an effective vaccine.

HIV-1 clade C (HIV-C) is the predominant subtype and is found in >56% of all HIV-1/AIDS cases worldwide (www.unaids.org). It is associated with the rapidly growing epidemics in populous regions, such as sub-Saharan Africa, India, and China, where B'/C recombinants with HIV-C *env* circulate. The epidemiological data imply an urgent need for vaccines to slow HIV-C spread.

Several reports have focused on the nature of recently transmitted HIV-C; these studies involved HIV-1 discordant heterosexual couples in Zambia that were followed prospectively [6], [7]. Viruses from newly infected individuals were more sensitive to neutralization by plasma of their chronically infected partners than contemporaneous viruses isolated from the latter. The newly transmitted HIV-C strains contained shorter variable loops compared to HIV-C sequences in the database [6], [7]. However, newly transmitted HIV-C strains overall mostly resemble typical primary HIV-C isolates with tier 2 neutralization phenotypes, which was also the case in a recent report by Seaman et al. [8], where 9 out of 11 relatively recently transmitted HIV-C isolates (Fiebig *≤* IV; [9]) were classified as tier 2 strains, 2 out of 11 as tier 1 and none as neutralization-resistant tier 3 strains. All of these 11 relatively recently transmitted HIV-C strains were linked to heterosexual transmission. Together, the data imply that heterosexual mucosal HIV-C transmission events involve viral strains with envelopes that exhibit at least some level of neutralization sensitivity in order to establish chronic infection in the new human host. Therefore, the availability of primate R5 SHIV challenge viruses that encode tier 1 as well as tier 2 primary HIV-1 envelopes will be useful for efficacy testing of candidate AIDS vaccines in macaque models.

There is general agreement that an AIDS vaccine should induce cellular as well as neutralizing antibody (nAb) responses, but inducing the latter at sufficiently high titers and breadth to neutralize typical primary HIV isolates has proven to be a daunting task. We suggest that development of vaccines designed to induce anti-HIV nAbs should proceed in a stepwise approach: preclinical vaccine efficacy studies in primates should first attempt to induce protection against simian-human immunodeficiency viruses (SHIVs) encoding primary HIV envelopes that are relatively easy to neutralize (tier 1 strains). Once this goal has been achieved, immunogens should then be optimized to induce protection against the more difficult-to-neutralize tier 2 viruses that exhibit neutralization sensitivity profiles of typical primary HIV isolates.

We have previously generated R5 clade C SHIV (SHIV-C) strains [1], [10], [11]. To construct the parental infectious molecular clone SHIV-1157i, *env* from the 6-month-old Zambian infant 1157i born to an HIV-C-positive mother was inserted into the SHIV-vpu^+^ backbone. During prospective long-term follow-up, the Zambian infant 1157i turned out to be a long-term non-progressor (LTNP) who has remained asymptomatic [12]. The rhesus monkey (RM)-adapted strain, SHIV-1157ip, is pathogenic and has caused AIDS in several monkeys thus far [10], but the rate of disease progression is relatively slow. The “early” SHIV-1157ip isolate, which had been adapted to RM through rapid serial passage, was significantly more sensitive neutralization in an earlier study [13] when compared to SHIV-1157ipd3N4 [1] the “late” virus version. The latter is an infectious molecular clone generated by directly cloning the 3′ half of the provirus from peripheral blood mononuclear cells (PBMC) of the first RM after it had progressed to AIDS. We also engineered the 3′ long terminal repeat of SHIV-1157ipd3N4 to contain an extra NF-κB site to accelerate viral replication in response to host cytokines, especially tumor necrosis factor (TNF)-α [1]. This “late” virus is still exclusively R5 tropic; it has a tier 2 neutralization phenotype [10], [11].

Here we report the construction of a tier 1 R5 SHIV, termed SHIV-1157ipEL, which is a chimera of the “early”, neutralization-sensitive SHIV-1157ip envelope and the “late”, engineered backbone of SHIV-1157ipd3N4 [1]. The latter is clearly a neutralization escape variant that evolved in the first RM recipient of the parental infectious molecular clone. SHIV-1157ipEL was rapidly passaged through four Indian RM. The passaged virus, SHIV-1157ipEL-p, was re-isolated from the last recipient within two weeks of inoculation and characterized. This new virus, a biological isolate, is exclusively R5-tropic, mucosally transmissible, and causes T-cell depletion in different anatomical compartments during acute infection. Furthermore, SHIV-1157ipEL-p is highly sensitive to neutralization in contrast to the late virus, SHIV-1157ipd3N4. Molecular modeling of the early and late Env-C proteins suggests that the orientation of the V2 loops may account for the differential susceptibility of the early and late Env forms to neutralization.

## Results

### The “late” SHIV-1157ipd3N4 is an escape variant

**Figure 1:**
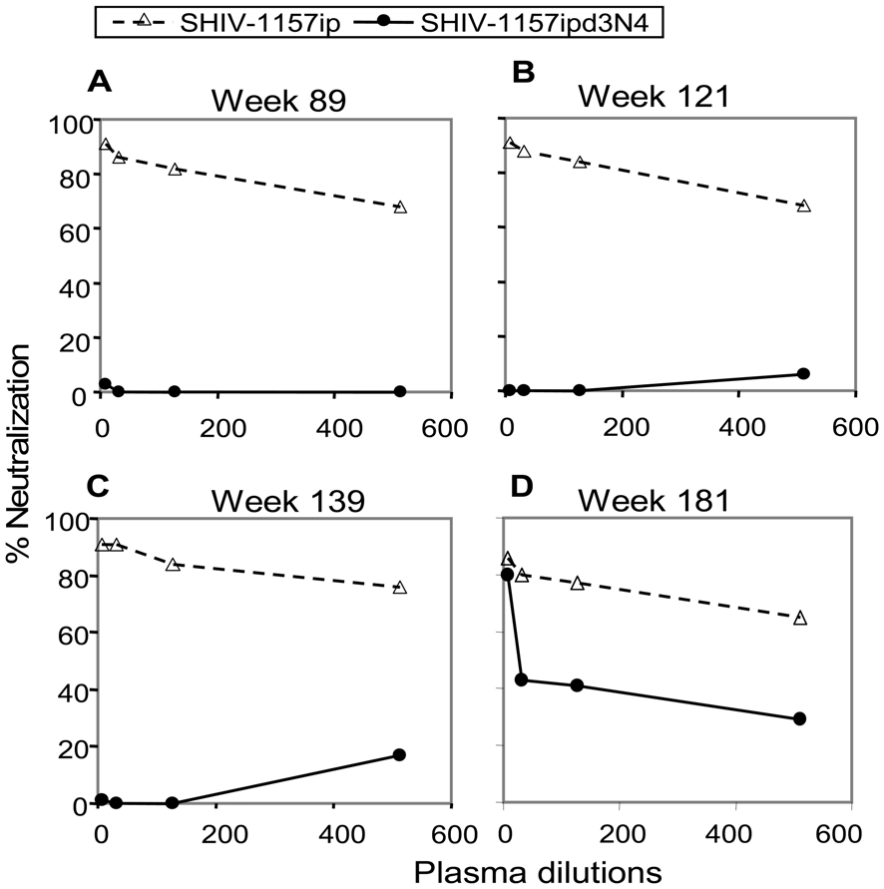
Selection of a neutralization escape variant in rhesus monkey RPn-8. This monkey was inoculated initially with an “early” form of the virus (the parental infectious molecular clone, SHIV-1157i; see Table 1), and 4 weeks after RPn-8 had progressed to AIDS (∼2.7 years), the “late” virus, SHIV-1157ipd3N4 [1], was generated by molecular cloning of the 3′ proviral half from RPn-8 PBMC DNA. Neutralization of SHIV-1157ip (“early” strain, open triangles) and SHIV-1157ipd3N4 (“late” strain, closed circles) with autologous plasma from animal RPn-8 at the time points post-inoculation indicated is shown (A–D), including contemporaneous plasma for the “late” virus collected at week 139 (panel C). Virus (grown in RM PBMC) was incubated with different dilutions of monkey plasma for 1 h before being added to TZM-bl cells in the presence of DEAE-dextran. Luciferase expression was measured on day 2 post-infection.

The “late” SHIV-1157ipd3N4 was isolated from monkey RPn-8 shortly after this animal developed AIDS (ca. 2.7 years post-inoculation). We compared the ability of autologous plasma samples collected from this host animal at various time points to neutralize SHIV-1157ip (early virus) and SHIV-1157ipd3N4 (late virus) using the TZM-bl cell assay [7], [14], [15] (Methods). Although the “early” SHIV-1157ip was effectively neutralized by all plasma samples of RPn-8 from weeks 89 to 181 (Fig. 1), this RM had persistently high levels of viremia throughout its course of disease progression. In contrast, “late” virus was not neutralized by early and contemporaneous plasma samples; only plasma from the latest time point (week 181) showed low-level neutralization. These autologous neutralization data suggest that the “late” SHIV-1157ipd3N4 arose as a neutralization escape variant in host RPn-8 by selective pressure exerted by high-level nAbs.

### Construction of a SHIV-1157ipEL encoding *env* from an African infant

**Figure 2:**
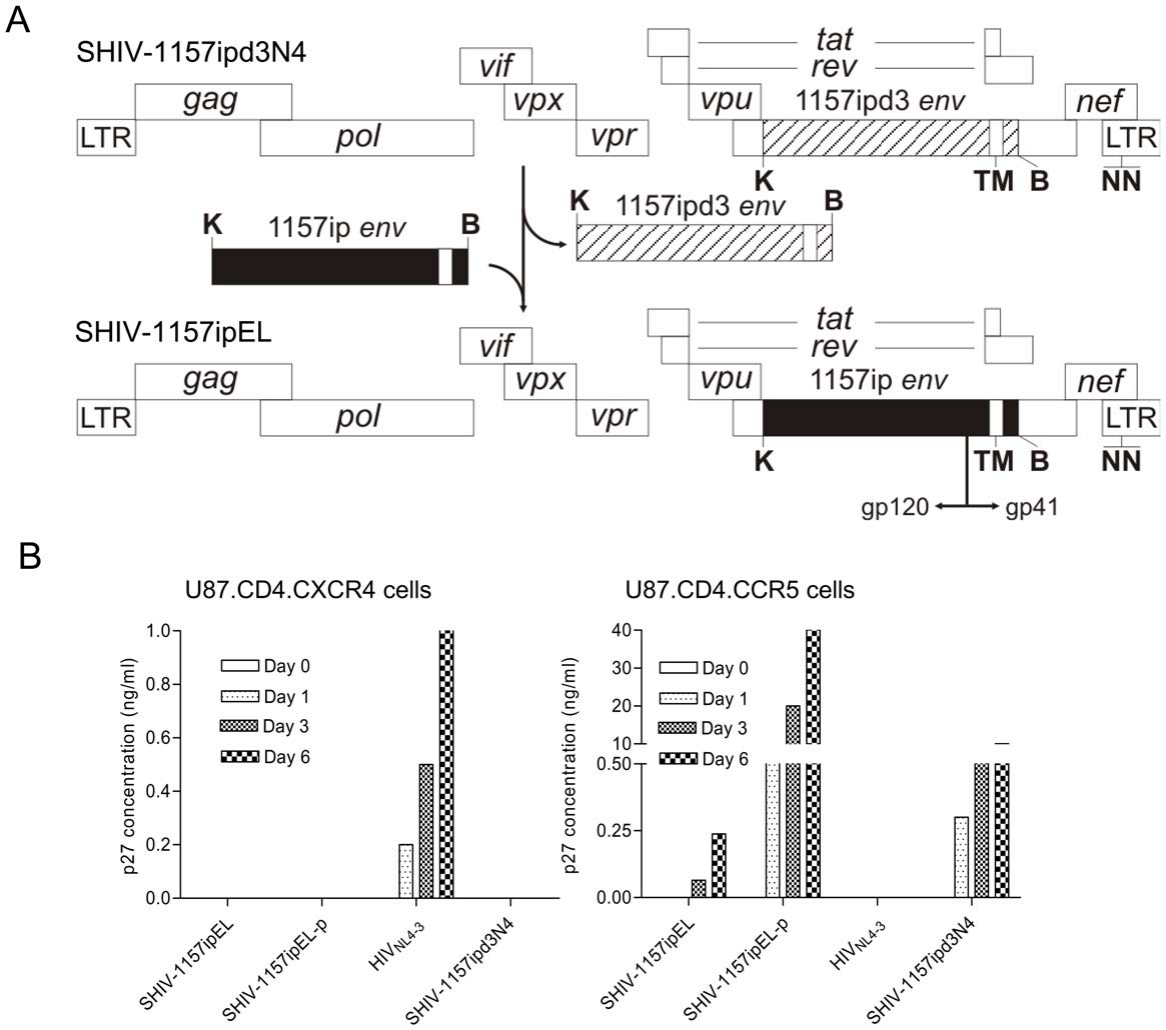
Construction and co-receptor usage of SHIV-1157ipEL. A. SHIV-1157ipd3N4 [1] was used as backbone to construct SHIV-1157ipEL. The 2.2 kb KpnI (K) - BamHI (B) fragment of the “early” SHIV-1157ip (spanning most of gp120, the entire gp41 extracellular domain, the transmembrane region (TM), and part of the cytoplasmic domain) (closed black bar) was used to replace the corresponding region of the proviral backbone (stippled bar). The modified 3′-half of was ligated with the 5′ half of SHIV-1157ipd3N4 DNA to form full-length SHIV-1157ipEL. NN: 2 NF-κB sites present in the 3′LTR. During viral replication, this duplication of NF-κB sites copied into the 5′LTR. B. Coreceptor usage of SHIV-1157ipEL and SHIV-1157ipEL-p. U87.CD4.CCR5 and U87.CD4.CXCR4 cells were exposed to parental SHIV-1157ipEL, passaged SHIV-1157ipEL-p, SHIV-1157ipd3N4 (clade C, R5 SHIV), and HIV_NL4-3_(HIV clade B *env,* X4). The levels of p27 Gag were measured in the supernatants as indicated.

The “late” infectious molecular clone, SHIV-1157ipd3N4, replicates to high levels and is transmissible across all mucosal surfaces tested [16]. However, it is relatively difficult to neutralize by active vaccination, e.g., using trimeric gp160 [13]. We sought to generate a neutralization-sensitive R5 SHIV-C version with the same optimized backbone present in SHIV-1157ipd3N4 (Table 1). SHIV-1157ip [10], an early biological isolate obtained after rapid passage through five RM, was used as source for an “early” HIV-C *env*. Full-length *env* genes were PCR-amplified from DNA isolated from co-cultured PBMC of a SHIV-1157ip-infected RM and were cloned into the expression vector pcDNA6/B. SHIV-1157ipEL was constructed using the late stage virus, SHIV-1157ipd3N4 [1], as the backbone (Fig. 2A). After exchanging *env*, the resulting chimera, SHIV-1157ipEL, contained most of gp120, the entire extracellular domain, the transmembrane region, and part of the cytoplasmic tail of gp41 of the early *env* gene. The initial cell-free SHIV-1157ipEL clone stock was generated by transfecting 293T cells with infectious proviral DNA and harvesting supernatant. The stock was tested for infectivity in TZM-bl cells [7], [14], [15], which express luciferase and β-galactosidase under the control of HIV-1 LTR (data not shown). The various R5 SHIV-C strains and their derivations are summarized in Table 1.

**Table 1:** Summary of R5 SHIV-C strains encoding an HIV1157i-related envelope.

Strain	Nature of strain	# NF-κB ites/LTR	Derivation	Neutralization sensitivity	Reference
SHIV-1157i	parental infectious molecular clone; not yet adapted to rhesus monkeys	1	result of initial molecular cloning, given to recipient monkey RPn-8, which developed AIDS		[1], [10]
SHIV-1157ip	passaged biological isolate; pathogenic	1	early virus isolated from monkey Kl-8 (passage 4)	tier 1	[10]
SHIV-1157ipd3N4	infectious molecular clone; pathogenic	2	late-stage virus generated by cloning the 3′ proviral half from infected PBMC of monkey RPn-8 4 weeks after the onset of AIDS (week 127 post-inoculation). RPn-8 had received the parental, non-passaged clone, SHIV-1157i.	tier 2	[1]
SHIV-1157ipEL	infectious molecular clone	2	“early-late” chimera generated by swapping *env* in the late SHIV-1157ipd3N4 with “early” *env*; non-passaged clone	tier 1	
SHIV-1157ipEL-p	biological isolate	2	passaged chimera reisolated from monkey ROm-8 (passage 3) at week 2 post-inoculation	tier 1	

Next, we assessed co-receptor usage of SHIV-1157ipEL with late stage virus SHIV-1157ipd3N4 and clade B NL4-3 virus as controls. SHIV-1157ipEL did not replicate in any cell line that lacked CCR5 (Fig. 2B; Methods). Productive infection was observed only in U87.CD4.CCR5 cells (Fig. 2B), suggesting that SHIV-1157ipEL uses CCR5 as an exclusive co-receptor for entry.

### Replication of SHIV-1157ipEL in RM PBMC

**Figure 3:**
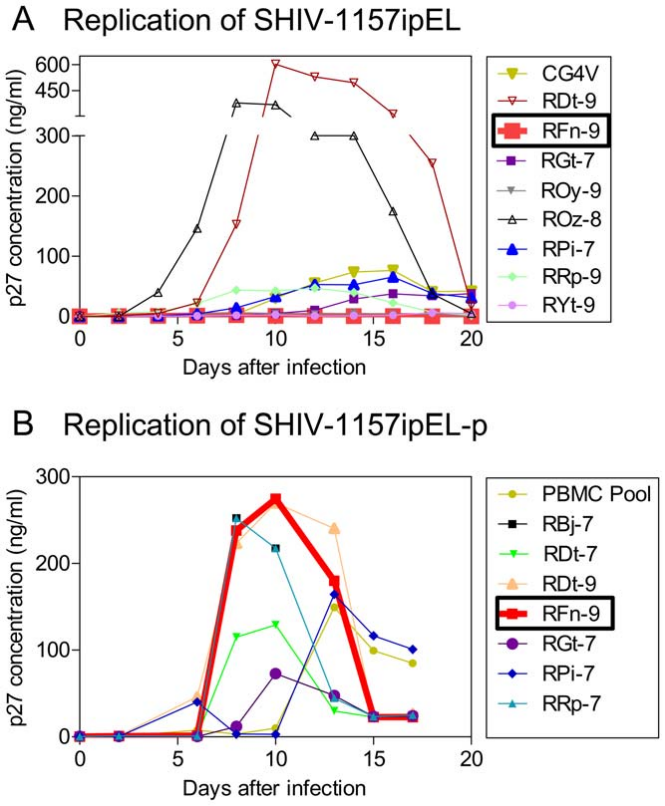
Replication of SHIV-1157ipEL and SHIV-1157ipEL-p in rhesus macaque PBMC. (A) PBMC from randomly selected naïve RM donors were stimulated with concanavalin A (Con-A) and exposed to SHIV-1157ipEL-containing supernatant (Methods). The PBMC of RM RFn-9 (thick red line) did not support the replication of parental SHIV-1157ipEL. (B) PBMC from random donors were stimulated with Con-A and exposed to passaged virus, SHIV-1157ipEL-p. Supernatants were harvested and p27 levels were measured at the time points indicated. PBMC cultures of RFn-9 (previously unable to support the replication of parental virus, see under A) now yielded high levels of p27 (thick red line), indicating SHIV-1157ipEL-p adaptation to RM. For RM listed in both A and B, aliquots of frozen PBMC from the same blood collection as in A were used for assays in B.

Our aim was to create a virus that would replicate reliably in all unselected, randomly chosen animals of the test species, rhesus macaques. Since it is well established that the ability of a primate lentivirus to replicate in cultured PBMC is predictive of its ability to productively infect the donor monkey, we randomly selected nine RM as PBMC donors to evaluate the replication kinetics of parental SHIV-1157ipEL in vitro. Undiluted virus, generated by transfection of 293T cells with the infectious molecular clone and followed by collection of cell-free supernatants, was used for the screen. While the new chimera replicated vigorously in the PBMC of two of the donors, PBMC of donor monkey RFn-9 did not support viral replication (Fig. 3A); PBMC cultures of other donors also yielded only little virus. Clearly, parental SHIV-1157EL was not replication competent in all PBMC cultures from randomly selected donor monkeys. This indicated a need to proceed with the in vivo adaption and the selection of progeny virus with better replication kinetics.

### Adaptation of SHIV-1157ipEL to RM and generation of SHIV-1157ipEL-p

**Figure 4:**
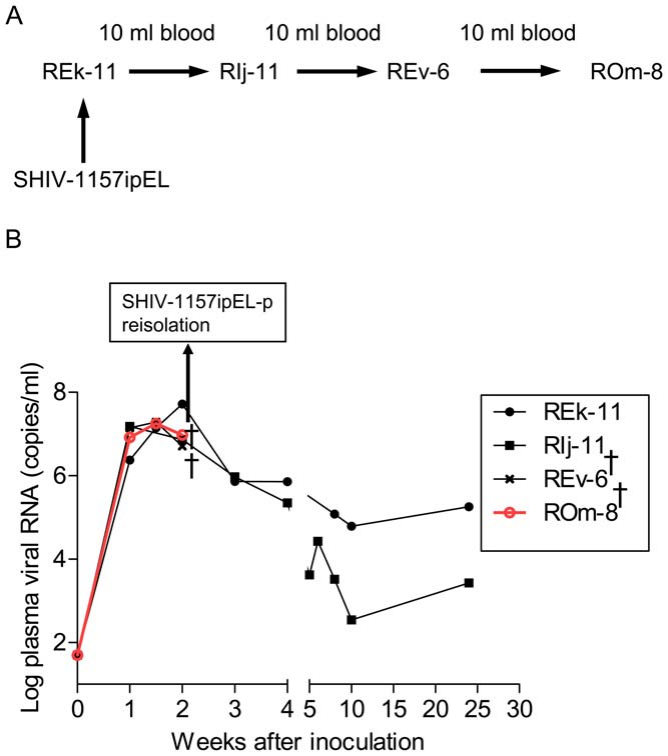
Serial passage of SHIV-1157ipEL in rhesus macaques. (A) Parental SHIV-1157ipEL was passaged rapidly in four Indian-origin RM through serial blood transfer at peak viremia (week 2). (B) Viral loads were measured after serial passage at the time points indicated. †, these RM were euthanized at peak viremia.

SHIV-1157ipEL was passaged through four RM (Fig. 4A). To avoid selection of a neutralization escape virus, we used rapid animal-to-animal passage of whole blood at the time of peak viremia (week 2 post-inoculation) to adapt the new SHIV strain to RM [10], [11]. The first macaque, REk-11, was inoculated intravenously (i.v.) with 10 ml of SHIV-1157ipEL stock (prepared in RM PBMC that were exposed to the cell-free supernatant of 293T cells transfected with proviral DNA); peak viremia reached 5.2×10^7^ RNA copies/ml at week 2 post-inoculation (Fig. 4B). Three additional animals were subjected to serial blood transfer (Fig. 4A). Passaged virus reached high peak viremia levels in all recipients, all of which seroconverted (data not shown). We reisolated virus at week 2 post-inoculation from the final recipient, ROm-8. The resulting passaged virus, SHIV-1157ipEL-p, an uncloned biological isolate, was expanded in RM PBMC.

### Replication of SHIV-1157ipEL-p in RM PBMC and co-receptor usage

Next, we assessed co-receptor usage by SHIV-1157ipEL-p. Productive infection was only seen with the CCR5-expressing cell line (Fig. 2B), suggesting that SHIV-1157ipEL-p maintained its exclusive R5 tropism after rapid animal-to-animal passage. We also tested whether SHIV-1157ipEL-p could grow in the PBMC of the RM that had previously failed to support the replication of non-adapted virus as well as in PBMC of other randomly selected donors. Adapted SHIV-1157ipEL-p replicated vigorously in all PBMC cultures tested, including PBMC from donor RFn-9 that had not supported the growth of the parental infectious molecular clone, SHIV-1157EL (Fig. 3A,B, boxed name), indicating its successful adaptation to RM.

### Phylogenetic analysis

Primary full-length *env* genes were PCR amplified from genomic DNA isolated two week post-inoculation from the last recipient (ROm-8) and cloned into the expression vector pcDNA6/B. As described previously [11], the *env* genes of the newly created SHIV strains clustered with HIV-C in a phylogenetic tree. Among the strains tested, the closest relationship was found with another set of SHIV-Cs that was derived from a pediatric HIV-C strain isolated from an infant belonging to the cohort of HIV-infected mothers/infants that are being followed prospectively at the University Hospital in Lusaka, Zambia.

### Evolution of SHIV-1157ipEL-p Env during adaptation

Sequence analysis was performed on the SHIV-1157ipEL-p *env* gene, which was cloned from genomic DNA of PBMC from ROm-8, the last RM recipient during the adaption process. Env DNA amplified from PBMC of ROm-8 (2 weeks after blood transfer) showed only three point mutations throughout gp160 relative to the parental (SHIV-1157ipEL) Env sequence (data not shown). This included loss of N295, a key amino acid residue in the epitope of the broadly reactive neutralizing antibody 2G12. Not surprisingly, the loss of N295 caused SHIV-1157ipEL to become resistant to 2G12 (data not shown). The absence of the 2G12 epitope in many primary HIV-C isolates is well known [7].

### Neutralization tier profiles

Primary HIV or SHIV strains are classified as tier 1 or tier 2 depending on their sensitivity to neutralization [17]. Tier 1 strains are markedly neutralization-sensitive; tier 2 strains are more difficult to neutralize and represent the average neutralization sensitivity of primary HIV isolates including most recently transmitted strains of HIV-C [7]. SHIV-1157ipEL and the passaged virus, SHIV-1157ipEL-p, were tested by TZM-bl assay against a panel of human neutralizing monoclonal antibodies (nmAbs) and polyclonal sera collected from HIV-positive individuals. Table 2 shows that the parental molecular clone, SHIV-1157ipEL, and the passaged biological isolate, SHIV-1157ipEL-p, are both tier 1 viruses, while the corresponding “late” virus, SHIV-1157ipd3N4, is categorized as tier 2 [1]. The fact that the neutralization profiles of SHIV-1157ipEL and SHIV-1157ipEL-p are similar indicates that our rapid adaptation strategy, whereby infected blood was transfused into a new recipient every two weeks, successfully avoided the outgrowth of a neutralization escape variant. Another R5 clade C SHIV, SHIV-2873Nip [11], was also classified as tier 2 strain. SHIV_SF162P3_ and SHIV_SF162P4_, previously classified as tier 2 and tier 1 viruses, respectively, were used as reference strains in the assay.

**Table 2:** Sensitivity of R5 SHIV strains to soluble CD4, human nmAbs and serum samples.

	*IC_50_ (µg/ml) in TZM-bl cells^a^*						*IC_50_ (reciprocal serum dilution) in TZM-bl cells^a^*							
*SHIV strain*	sCD4	IgG1 12	2G12	2F5	4E10	HIVIG	BB47	BB55	BB68	BB75	BB80	BB81	BB87	Tier
Clade C														
SHIV-1157ipEL (early/late chimera mol. clone)	1.7	0.7	>25	>25	>25	88.6	4,274	3,286	666	1,245	1,822	691	3,278	1
SHIV-1157ipEL-p (early/late chimera; RM passaged)	0.5	1.4	>25	>25	>25	150.8	1,193	1,792	653	824	1,135	427	2,707	1
SHIV-1157ipd3N4^b^ (late isolate)	0.4	7.0	>25	>25	>25	1,160	105	131	86	79	72	47	260	2
SHIV-2873Nip b (early isolate)	1.5	>25	>25	>25	>25	>2,500	<20	70	266	66	56	<20	25	2
Clade B														
SHIV_SF162P4_ b	0.2	<0.01	15.7	1.5	0.7	25	363	2,543	328	615	828	211	3,603	1
SHIV_SF162P3_ b	11.6	6.0	>25	>25	>25	1,505	24	65	22	27	25	<20	180	2

Spectrum of neutralization sensitivity of R5 SHIV strains encoding HIV clade B or C *env*. SHIV-C strains were grown in human PBMC.

a values represent the concentration (µg/ml for soluble CD4 (sCD4) and human nmAbs IgG1b12, 2G12, 2F5, 4E10, or HIVIG) or the dilution (for serum samples) at which relative luciferase units (RLU) were reduced 50% compared to virus control wells. BB47, BB55, BB68, BB75, BB80, BB81, and BB87 are serum samples from individuals infected with HIV-1 clade C. HIVIG, polyclonal high-titer anti-HIV Ig preparation.

b data previously published [11] and given as basis of comparison.

### SHIV-1157ipEL-p is efficiently neutralized by autologous and heterologous RM plasma/sera

For a challenge virus to be useful for anti-HIV-C vaccine development, it should maintain a neutralization-sensitive Env structure. We assessed SHIV-1157ipEL-p neutralization sensitivity in TZM-bl cell-based assays with autologous and heterologous RM plasma or serum samples collected from R5 SHIV-infected RM throughout the course of infection. As shown in Table 3, all animals tested developed high nAb titers against SHIV-1157ipEL-p but only weak or low titers against SHIV-1157ipd3N4. While these data are consistent with the high susceptibility of the “early” virus, SHIV-1157ipEL-p, to neutralization and compatible with its tier 1 phenotype, it is interesting to note that even sera from RM that were chronically infected with the “late” SHIV-1157ipd3N4 developed higher nAb titers against SHIV-1157ipEL-p than against SHIV-1157ipd3N4 itself. Importantly, sera from RM infected with the heterologous tier 2 SHIV-2873Nip also developed strong nAb titers against SHIV-1157ipEL-p and very low titers against SHIV-1157ipd3N4.

**Table 3:** Neutralization titers of autologous and heterologous polyclonal serum/plasma samples against SHIV-1157ipEL-p (early Env) and SHIV-1157ipd3N4 (late Env).

Plasma	R5 SHIV strain harbored by rhesus monkeys (RM) and animal name	Titers (serum/plasma dilution at 50% neutralization)	
		SHIV-1157ipEL-p	SHIV-1157ipd3N4
Autologous	SHIV-1157ip-infected RM (early virus)		
	RIj-11	88	<10
	REk-11	>640	<10
	RAo-8	560	32
	RKl-8	148	47
	RTs-7	93	20
	SHIV-1157ipd3N4-infected RM (late virus)		
	RQ3911	140	30
	RJs-10	110	23
	Rho-10	336	84
	RBg-9	40	36
Heterologous	SHIV-2873Nip-infected RM		
	RBg-10	90	18
	RUf-10	54	<10
	RNt-9	340	<10
	RTb-11	28	<10

Autologous plasma samples were collected from 6 months to 3 years post-inoculation; heterologous plasma samples were collected from 6 months to 1 year post-inoculation. SHIV-C strains were grown in RM PBMC.

### Sequence and structural analysis of SHIV-1157ipEL-p, SHIV-1157ipEL-pΔ3N and SHIV-1157ipd3N4

**Figure 5:**
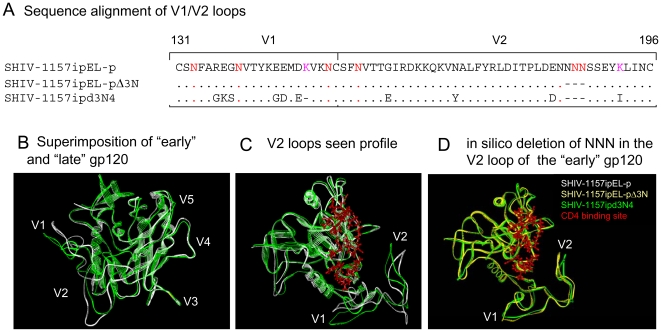
(A) Sequence alignment of V1/V2 sites of and SHIV-1157ipEL-p, SHIV-1157ipEL-pΔ3N and SHIV-1157ipd3N4 (numbering according to HXB2). (B) Molecular modeling of SHIV-1157ipEL-p and SHIV-1157ipd3N4 sequences was performed using the X-ray structure of the CD4-bound YU2 gp120 core [20]; PDB code 1RZK). The V1, V2, and V3 loops were modeled onto the core. Mutations inducing structural heterogeneity in the V1 and V2 loops were identified. (C and D) Illustration of three-dimensional (3D) gp120 of SHIV-1157ipEL-p (white), SHIV-1157ipd3N4 (green), and (D) SHIV-1157ipEL-pΔ3N (yellow) showing that access to the CD4 binding site (red) is more restricted for SHIV-1157ipEL-pΔ3N and SHIV-1157ipd3N4 than for SHIV-1157ipEL-p.

As SHIV-1157ipEL-p and SHIV-1157ipd3N4 share 90% sequence homology in Env but have different neutralization profiles, we sought to link structural features with differential neutralization sensitivity. First, we searched for potential N-linked glycosylation (PNG) sites present in both SHIV strains. Compared to SHIV-1157ipd3N4 (containing 25 PNG sites in gp120), SHIV-1157ipEL-p has an additional PNG site in the V2 loop; all other PNG sites are identical (Fig. 5A). This PNG site is adjacent to another one, and steric hindrance might prevent carbohydrate addition to both asparagines. Of note, many of the tier 1 HIV sequences contain contiguous PNG sites in the V2 loop [18]. Furthermore, we identified differences in the hypervariable regions of the two SHIVs. For instance, the V2 loop of SHIV-1157ipEL-p is *longer* than that of SHIV-1157ipd3N4 by three amino acids, namely three asparagines (NNN) at positions 185–187. Also, the V2 loop N170Y mutation of SHIV-1157ipd3N4 could be important for access to the CD4 binding site (CD4bs). Interestingly, SHIV-1157ipEL-p has one more basic amino acid in each of the V1, V2, and V3 loops than SHIV-157ipd3N4 (Fig. 5A), which may result in increased antibody binding to these loops [19].

Using the established core structure of HIV-1 YU2 Env [20], we performed molecular modeling to superimpose the gp120 sequences of SHIV-1157ipEL-p, and SHIV-1157ipd3N4 (Fig. 5B). The main differences between the sequences are located in the V2 loops, which are packed closer to the core of gp120 and the CD4 binding site in SHIV-1157ipd3N4, than in SHIV-1157ipEL-p (Fig. 5C). Interestingly, the shorter V2 loop of the “late” virus (Fig. 5C, shown in green) appears to occlude access to the CD4bs (shown in red), which would make it difficult for anti-CD4bs nmAbs to bind to their cognate epitopes. Furthermore, molecular modeling predicted that deletion of the NNN residues at positions 185–187 in the tier 1 SHIV-1157ipEL-p would tilt the V2 loop toward the CD4bs in a direction analogous to that of the tier 2 SHIV-1157ipd3N4 (Fig. 5D, yellow and green structures) and thus would likely obstruct access to the CD4bs.

**Figure 6:**
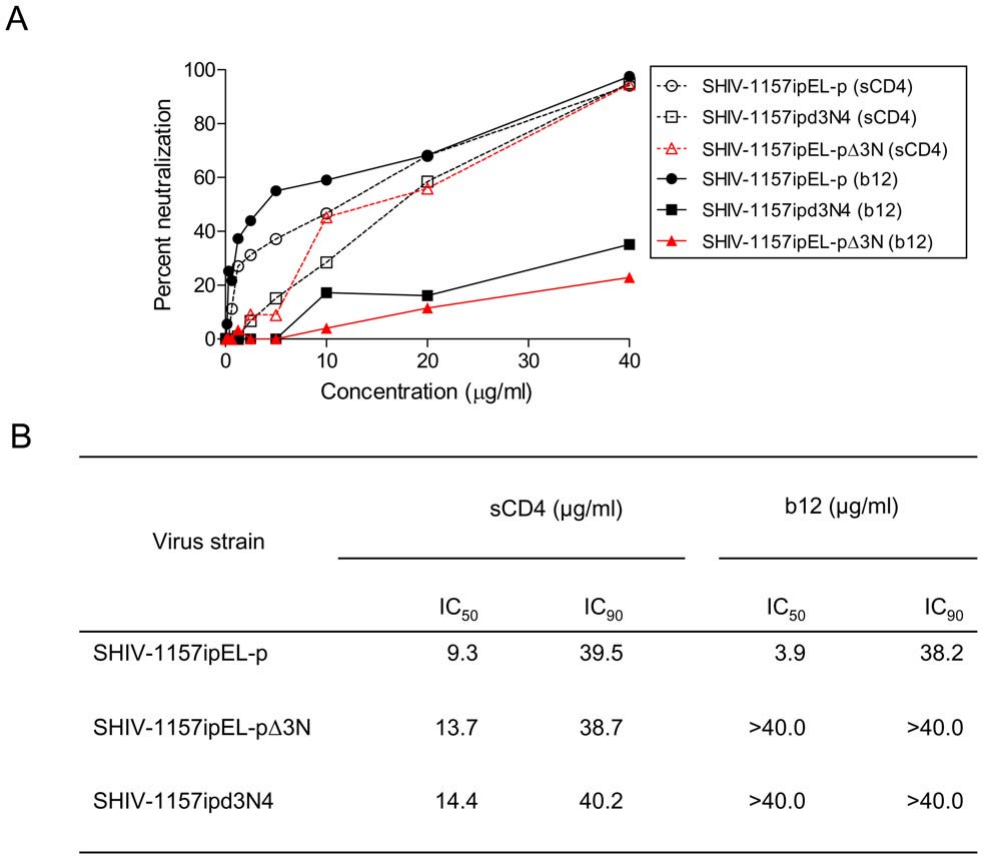
Neutralization of SHIV-1157ipEL-p, SHIV-1157ipEL-pΔ3N and SHIV-1157ipd3N4 by sCD4 and b12. The assays were performed in TZM-bl cells using virus stocks grown in RM PBMC. (A) Dotted lines, neutralization of SHIV-1157ipEL-p (circles), SHIV-1157ipEL-pΔ3N (red color with triangles) and SHIV-1157ipd3N4 (squares) using sCD4; solid lines, neutralization of the above SHIVs using the human nmAb b12. (B) Values represent the concentration (µg/ml) for sCD4 and human b12 at which relative luciferase units (RLU) were reduced 50% or 90% compared to virus control wells, respectively.

To test this notion, we used site-directed mutagenesis to construct the NNN-deletion mutant, termed SHIV-1157ipEL-pΔ3N (Fig. 5A & D). Next, we tested the neutralization of SHIV-1157ipEL-p, SHIV-1157ipEL-pΔ3N and SHIV-1157ipd3N4 by sCD4 and b12 in the TZM-bl assay (Fig. 6A). All three viruses exhibited similar neutralization sensitivity to soluble CD4 (sCD4). However, a major difference was observed for b12. Indeed, while this human anti-CD4bs nmAb neutralized SHIV-1157ipEL-p with an IC_50_ of 3.9 µg/ml, SHIV-1157ipEL-pΔ3N and SHIV-1157ipd3N4 were not neutralized even at the highest nmAb concentration tested (40 µg/ml; Fig. 6B). These findings imply that V2 loop orientation may be sufficient to change the neutralization profile by regulating the access to the CD4bs.

### Mucosal transmissibility of SHIV-1157ipEL-p

**Figure 7:**
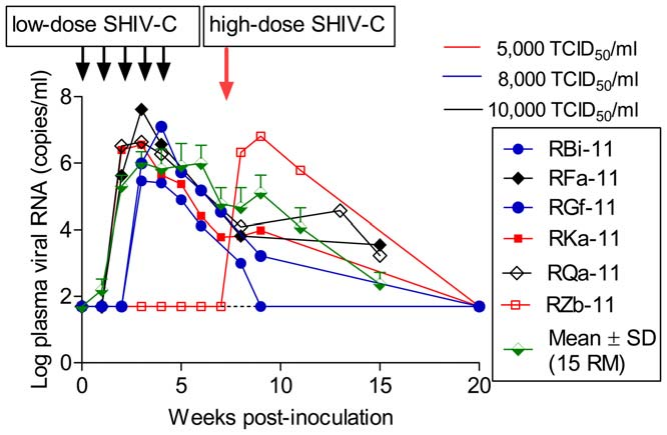
Intrarectal inoculation of SHIV-1157ipEL-p. Six monkeys were used in a repeated low-dose i.r. titration; the aim was to find a virus dose that resulted in systemic infection (defined as viral RNA ≥10^4^ copies/ml) after a maximum of five weekly i.r. inoculations. RM remaining uninfected at week 2 after the 5^th^ weekly low-dose virus challenge were given a single high-dose of SHIV-1157ipEL-p (1.5×10^5^ TCID_50_). The green half-diamonds represent mean ± SD of 15 additional RM used in unpublished vaccine efficacy studies as unvaccinated controls; these animals were given maximally five weekly i.r. inoculations at 8,000 TCID_50_. The horizontal dotted line indicates lower limit of detection (<50 viral RNA copies/ml).

Since mucosal transmission is a major mode for HIV acquisition in humans, candidate AIDS vaccines should be designed to protect against mucosal virus exposures. Thus, primate challenge models should test vaccine safety and efficacy also in relation to this route (reviewed in [21]). We generated a large stock of SHIV-1157ipEL-p in RM PBMC and determined its ability to cause systemic infection after repeated weekly, low-dose intra-rectal (i.r.) challenges using a maximum of five weekly inoculations. Of the two RM exposed to a weekly dose of 5,000 TCID_50_, one animal became viremic after three inoculations, while the second RM remained aviremic and was subsequently given a single dose of 1.5×10^5^ TCID_50_ that promptly resulted in high viremia (Fig. 7). Another two RM were challenged weekly with 8,000 TCID_50_; all became viremic after 3 or 4 inoculations. Lastly, two RM given 10,000 TCID_50_ became systemically infected after 2 or 3 weekly inoculations (Fig. 7). In the interim, SHIV-1157ipEL-p has been used in vaccine challenge studies using a maximum of five weekly inoculations with 8,000 TCID_50_ Overall, a total of 17 unvaccinated RM have been challenged with this regimen; 16 have developed systemic infection (94%), including two animals that received 8,000 TCID_50_ during the repeated low-dose titration.

### Signs of SHIV-1157ipEL-p pathogenicity

**Figure 8:**
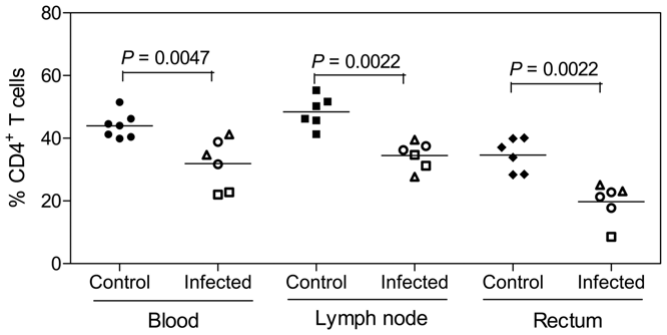
T-cell depletion during acute SHIV-1157ipEL-p viremia. The percent CD4^+^ T cells in blood, lymph node and gut was estimated compared with cells from naïve monkeys (closed symbols). Biopsy specimens were collected during the acute phase of infection. Two of the 2-week time points were obtained from last two i.v.-inoculated RM used for viral adaptation, the remainder were collected from RM undergoing the 5x weekly i.r. challenge-dose titration. We assigned peak viremia as being week 2 after the pen-ultimate i.r. inoculation, the one that most likely was successful in achieving systemic infection. Individual time points are shown with different symbols (open squares, week 2; open triangles, week 5; open circles, week 12).

Within 3–6 months of follow-up, all the SHIV-1157ipEL-p-infected monkeys have maintained absolute CD4 T-cell counts in peripheral blood of >500 cells/µl. However, from approximately 8 weeks post-inoculation onwards, peripheral blood CD4^+^ memory T cells (assessed by CD4^+^CD29^+^ double staining) have been persistently low (<10%) in all infected animals (data not shown). Depletion of CD4^+^CD29^+^ cells has been predictive of depletion of absolute CD4^+^ T-cell counts [10], [22]. Previous reports have shown that depletion of gut CD4^+^ T cells in acute infection is typical hallmark of HIV infection (reviewed in [23]). We therefore assessed the effect of SHIV-1157ipEL-p on rectal and lymph node lymphocytes during acute-stage infection (2–12 weeks post-inoculation). Compared to uninfected controls, the percentage of CD4^+^ T cells among the lymphocytes isolated from blood, lymph nodes, and gut in all the infected monkeys tested was significantly lower (*p*<0.05; Mann-Whitney test; Fig. 8).

## Discussion

We describe the development and characterization of the R5 SHIV-1157ipEL-p that encodes an early *env* from an African pediatric HIV-C isolate. We compared structural and neutralization profiles of the new SHIV-1157ipEL-p with that of a SHIV that encodes the late version of the same envelope, SHIV-1157ipd3N4. A number of distinctive features can be ascribed to SHIV-1157ipEL-p: 1) it carries *env* of an early, relatively recently transmitted HIV-C; 2) SHIV-1157ipEL-p contains additional NF-kB sites in the LTRs; 3) it contains the backbone of the late SHIV-1157ipd3N4, which also has a deletion in the 3′ end of HIV *env* that restored the original SIVmac239 *env* C-terminus allowing it to extend into the original SIV *nef* overlap region [1]; 4) SHIV-1157ipEL-p is exclusively R5 tropic; 5) it is highly sensitive to neutralization with a tier 1 profile; 6) it is mucosally transmissible and can be used to assess candidate AIDS vaccine efficacy against mucosal exposure; 7) signs of SHIV-1157ipEL-p pathogenicity in RM include memory T-cell depletion in blood, and loss of CD4^+^ T cells in PBMC, lymph node and gut during acute viremia; and 8) the HIV-C Env evolution in our SHIV-C-infected RM recapitulated patterns of molecular evolution observed in HIV-infected humans, including individuals harboring HIV-C [24],[25].

Genetic analysis of SHIV-1157ipEL-p *env* showed just three point mutations throughout the envelope when compared to the parental chimera SHIV-1157ipEL, indicating that our rapid, every two-week passage strategy resulted in only minor Env changes. Consistent with this finding is the maintenance of the tier 1 neutralization profile. In contrast, our tier 2 SHIV strains, SHIV-1157ipd3N4 and SHIV-2873Nip, showed extensive mutations and amino acid deletions throughout the gp160 when reisolated 2.7 and 1 years post-inoculation, respectively, when compared to the parental infectious molecular clones [25]. These more extensive mutations seen after long-term *in vivo* replication were associated with a change to a tier 2 profile for SHIV-1157ipd3N4.

SHIV-1157ipd3N4 and SHIV-1157ipEL-p are 90% identical in their Env amino acid sequences but differ markedly in their neutralization profiles. Previous reports demonstrated that modifications of N-glycosylation sites, variable loop lengths, charges of amino acid residues or a combination of these factors are responsible for differences in neutralization profiles [7], [19], [24], [25], [26], [27], [28], [29], [30], [31], [32], [33], [34], [35], [36], including data from clade B SHIV/macaque models [37], [38], [39], [40], [41], [42], [43]. We did not find any significant differences in the early and late SHIV-C Env sequences, with the exception of the loss of one PNG site in the V2 loop in SHIV-1157ipd3N4. Several studies have indicated that the majority of changes in charged amino acids occur in the V1/V2 loops of gp120 and affect neutralization sensitivity [42], [44], [45], [46], [47], [48], [49], [50]. In our study, differences in neutralization sensitivity were linked to the V1, V2 and V3 loops of viruses encoding “early” forms of the 1157ip envelopes containing disproportionately more basic amino acids compared to the “late” Env of SHIV-1147ipd3N4. Recently, Wu et al. [19] showed that increasing the number of positively charged amino acids in gp120 enhanced exposure of the CD4bs, which rendered virus more sensitive to nAbs. Together, these findings indicate that changes in charge could be an important determinant of neutralization sensitivity.

Interestingly, the “early” SHIV-1157ipEL-p Env has a longer V2 loop than the “late” SHIV-1157ipd3N4. This is in contrast to the observations of Li et al. [7], who reported that recently transmitted HIV-C strains had shorter variable loop regions compared to all HIV-C strains in the database. Others have postulated that variable loop length is an important determinant for neutralization sensitivity, and V1/V2 loops have been proposed to act as protective barriers around the CD4bs, masking its accessibility to nAbs, thus resulting in different neutralization profiles [47], [48], [50], [51], [52]. In our comparison of “early” and “late” forms of the HIV-C Env molecules in the 1157i series, we found that the V2 loop is packed closer to the CD4bs in the case of the “late” SHIV-1157ipd3N4, which probably restricted nAb access to the CD4bs, as reflected in the IC_50_ and IC_90_ values for nmAb b12. Changes in the positions of the variable loops have been linked to increasing neutralization resistance by Ye et al. [43], who analyzed Env mutations that arose when the clade B SHIV-HXBc2, which encodes a laboratory-adapted, neutralization-sensitive HIV *env,* was passaged in macaques to yield the highly pathogenic SHIV_KU-1_. The *env* gene of the latter was swapped with the “early” *env* gene in SHIV-HXBc2, giving rise to SHIV-HXBc2P 3.2, which not only caused rapid CD4+ T-cell depletion but was also significantly more neutralization resistant than the “early” SHIV-HXBc2. Antibody competition analysis, used to deduce structural differences between the two Env molecules, implied a close proximity of the V2 and V3 loops to the gp120 core and the CD4bs, thus restricting access of nAbs and soluble CD4 to the CD4bs. Of note, the molecular modeling we employed here to analyze HIV-C Env mutations arising during the course of SHIV-C infection arrived at same conclusion that increasing neutralization resistance is linked to the position of V2 with regards to the CD4bs. In fact, molecular modeling predicted that the short NNN deletion in V2 of the tier 1 virus, SHIV-1157ipEL-p, might be sufficient to hinder access to the CD4bs. This prediction was confirmed experimentally with an isogenic virus that differed only in the NNN residues at positions 185–187 from the “early” virus: sensitivity of the two viruses to b12 was very different as only SHIV-1157ipEL-p was susceptible to this nmAb. Therefore, our current results on SHIV-C constructs in RM emphasize the importance of V2 loop position relative to the CD4bs in determining neutralization sensitivity.

Two recent studies showed that different pathways were involved in the neutralization escape of early forms of HIV-C from autologous antibodies in South African individuals [24], [25]. As expected, later viruses were more difficult to neutralize by contemporaneous autologous sera. In one study, HIV-C neutralization escape was driven by modifications in V1/V2 or the alpha-2 helix of C3 [24], whereas Rong et al. [25] noted that changes in V1 to V5 with involvement of V1 and gp41 regions predominated during HIV-C Env evolution in their study subjects. In our case, a few changes in V2 between SHIV-1157ipEL-p and SHIV-1157ipd3N4 could explain the difference in neutralization profiles, which is one of the sites associated with HIV-C neutralization escape in infected humans. Interestingly, monkeys challenged with “late” SHIV-1157ipd3N4 had much higher neutralization titers against the early form, SHIV-1157ipEL-p, than the autologous late SHIV-1157ipd3N4. Taken together, our SHIV-C data suggest that HIV-C envelope evolution that occurred under the selective pressure of RM nAbs displays some of the hallmarks of HIV-C Env evolution in seen in chronically infected humans.

SHIV-1157ipEL-p infection resulted in high peak viral RNA loads and signs of pathogenicity during acute infection. Studies with SIV-infected RM and HIV-infected humans document that acute infection is accompanied by a marked depletion of CD4^+^ memory T cells primarily in mucosal tissues [53], [54], [55]. We observed loss of memory CD4^+^CD29^+^ T cells in peripheral blood and a depletion of CD4^+^ T cells in the blood, lymph nodes and gut tissues of SHIV-1157ipEL-p-infected RM. This pattern resembles the one reported in acutely HIV-infected humans [53], [54], [55]. Together, our in vivo data indicate that SHIV-1157ipEL-p is highly replication-competent and exhibits early signs of pathogenicity. Prolonged prospective follow-up will reveal the full spectrum of disease associated with this R5 SHIV-C strain. Of note, both SHIV-1157ip, the donor of the *env* gene, and SHIV-1157ipd3N4, the donor of the backbone, are pathogenic and have induced AIDS within 3 years [10], [16]. We expect that SHIV-1157ipEL-p-infected RM will progress to AIDS, since we used the late backbone and the early-passage *env* to build this chimera.

SHIV-1157ipEL-p was classified as a tier 1 virus based on its high susceptibility to neutralization. This property allows for its use as a challenge virus for nAb-response-based AIDS vaccine trials in RM. In fact, it is the only R5 SHIV-C tier 1 virus described to date. Although we have previously reported the construction and biological properties of two R5 SHIV-C strains, SHIV-1157ipd3N4 and SHIV-2873Nip, both were determined to be tier 2 viruses with neutralization sensitivity profiles typically seen in primary HIV isolates. In addition to our matched tier 1/tier 2 R5 SHIV-Cs based upon envelopes from HIV1157i, another tier 1/tier 2 pair of R5 SHIVs has been described, which are based upon the R5 envelopes of HIV_SF162_, a clade B strain. The parental SHIV_SF162_ gave rise to SHIV_SF162P3_/SHIV_SF162P4_ during sequential blood/bone marrow transfers from an infected RM donor to new recipients [56], [57], [58]; SHIV_SF162P3_ and SHIV_SF162P4_ differ in their neutralization sensitivities and are classified as tier 2 and tier 1 strains, respectively. Interestingly, the tier 1 strain arose in an animal from a later passage compared to the tier 2 SHIV_SF162P3_ [56]. In contrast, the envelope in our “late” SHIV-1157ipd3N4 represents a neutralization escape variant of the early Env version.

Since induction of high nAb levels with extended breadth is a major hurdle in developing an effective AIDS vaccine and current immunogens have generally not induced nAbs capable of neutralizing heterologous tier 2 viruses in vitro, it is informative to evaluate candidate anti-HIV-C AIDS vaccines by testing efficacy with a tier 1 R5 SHIV-C challenge virus in primate models when tier 2 challenge viruses are not neutralized in vitro. Once substantial protection is achieved against a tier 1 challenge virus, the subsequent steps in vaccine development would involve immunogen optimization and then employ tier 2 R5 SHIV challenge strains, such as SHIV-1157ipd3N4 or SHIV-2873Nip [11]. Of note though, our results revealed an intriguing finding: monkeys infected with the late, tier 2 SHIV-1157ipd3N4 showed equal or better neutralization titers for the tier 1 isolate SHIV-1157ipEL-p, even though these animals were never exposed to the early version of this HIV-C Env. This finding may need to be taken into account for the design of effective immunogens against tier 1 and tier 2 isolates.

In summary, with the use of our R5 SHIV-C constructs in RM, SHIV-1157ipEL-p, SHIV-1157ipd3N4 and related viruses, we have shown that natural evolution of Env sequences was predominantly associated with changes in the V2 loops, which influenced neutralization sensitivity profiles. As such, the molecular evolution of HIV-C Env in the context of a pathogenic R5 SHIV in rhesus monkeys recapitulated HIV-C Env evolution in humans. SHIV-1157ipEL-p and its tier 2 counterpart, SHIV-1157ipd3N4, will be biologically relevant tools to evaluate vaccine candidates designed to induce anti-HIV-C nAb responses in primate model studies.

## Materials and Methods

### Original virus isolates and nomenclature

SHIV-1157i represents the initial infectious molecular clone that encodes *env* of a primary HIV-C, HIV1157i, isolated from the relatively recently infected Zambian infant 1157i [1], [10]; “i” indicates a virus strain (or *env* gene) isolated from a human infant. SHIV-1157ip is the initial RM-adapted biological isolate (“p” designates a passaged, i.e., monkey-adapted form) [10] that was pathogenic but caused disease slowly [10] (Table 1). To generate a more replication-fit virus, an infectious molecular clone was generated by directly cloning a virus from a SHIV-1157i-infected RM after it had developed AIDS 2.7 years post-inoculation. The resulting SHIV-1157ipd3N4 clone was also engineered to contain two NF-κB sites per LTR, instead of the usual single NF-kB site that is present in the LTR of SIVmac239, the backbone used to generate the SHIV constructs. The late-stage SHIV-1157ipd3N4, however, was relatively difficult to neutralize. SHIV-1157ipEL, an infectious molecular clone, represents a chimera generated by inserting an early form of the SHIV-1157ip *env* into the backbone of the late SHIV-1157ipd3N4. SHIV-1157ipEL-p is a biological isolate obtained after a short, serial passage of SHIV-1157ipEL through four RM and isolated from the last RM (ROm-8) two weeks post-inoculation (Table 1).

### Construction of SHIV-1157ipEL molecular clones

SHIV-1157ip, an early biological isolate, was PCR-amplified using high-fidelity DNA polymerase and the following primer pairs:5′GGGGGAAGCTTATGAGAGTGATGGGGATACAGAGG-3′ and 5′-CCCCCTCGAGTTATTGCAAAGCTGCTTCAAAGCCC-3′. The full-length SHIV-1157ip *env* was digested with restriction enzymes *Hind*III and *Xho*I and cloned into the vector pcDNA6/myc-His B (Invitrogen, Carlsbad, CA). After digestion with *Kpn*I (K)-*BamH*I (B), a 2.2 kb fragment of SHIV-1157ip *env* (spanning most of gp120, the entire gp41 extracellular domain, the transmembrane region (TM), and part of the cytoplasmic domain) was obtained and used to replace the corresponding region of SHIV-1157ipd3N4 *env*. The modified 3′-half was ligated with the 5′-half of SHIV-1157ipd3N4 [1] proviral DNA to generate full-length SHIV-1157ipEL.

### Co-receptor usage of SHIV constructs

The U87 or GHOST cell lines expressing CD4 alone or CD4 and HIV-1/SIV co-receptors, obtained from the NIH AIDS Research & Reference Reagents Program (ARRRP, Germantown, MD), were used to study viral tropism. U87.CD4, U87.CD4.CCR1, U87.CD4.CCR2, U87.CD4.CCR3, U87.CD4.CXCR4, U87.CD4.CCR5, GHOST.BOB, and GHOST.BONZO were infected with stocks of SHIV-1157ipEL and SHIV-1157ipEL-p. SHIV-1157ipd3N4 (clade C, R5) and NL4-3 (clade B, X4) were used as controls. Cells were washed and resuspended in 1 ml of fresh medium. Supernatants were collected on days 0, 1, 3, and 5, for estimation of p27.

### Replication kinetics in rhesus macaque PBMC

PBMC from randomly selected naïve RM donors were stimulated with concanavalin A (Con-A) (5 µg/ml), followed by the addition of interleukin-2 (IL-2) (20 U/ml) for 72h. PBMC were washed twice and exposed to supernatant containing SHIV-1157ipEL or SHIV-1157ipEL-p viruses and incubated for 2 h at 37°C. PBMC were washed twice and added to wells at 2×10^6^/well in 12-well plates. Starting from day of exposure, 200 µl of the medium were harvested and replaced every second day with fresh medium (containing IL-2) up to day 20 and assayed for p27 levels.

### Animals and animal care

RM (*Macaca mulatta*) of Indian origin were housed at the Yerkes National Primate Research Center (YNPRC, Emory University, Atlanta, GA) according to U.S. Public Health Service/National Institutes of Health guidelines on the care and use of laboratory animals that include recommendations for animal welfare and procedures to ameliorate suffering of non-human primates. YNPRC facilities are fully accredited by the Association for Assessment and Accreditation of Laboratory Animal Care International. Animal experiments were approved by the Institutional Animal Care and Use Committees of 1) YNPRC on 11/21/2008 and continuing approval on 11/21/2009 (protocol # 261-2008Y), and 2) the Dana-Farber Cancer Institute via a Collaborating Institution Animal Use Agreement.

### Serial passage of SHIV-1157ipEL

RM REk-11 was inoculated i.v. with 10 ml of a SHIV-1157ipEL stock prepared from RM PBMC. After REk-11 was confirmed to be virus-positive as assessed by real-time RT-PCR [59] at week 1 post-inoculation, 10 ml of blood from this RM was transferred i.v. to monkey RIj-11. Serial blood transfer was performed at 2 weeks post-inoculation, during the expected time of peak viremia (Fig. 4A). All animals were monitored for viral loads, antibody responses, and T-cell subsets.

### Measurement of plasma viral RNA levels

Plasma viral RNA was isolated by use of the QiaAmp Viral RNA Mini-Kit (Qiagen) and viral RNA levels were measured by quantitative reverse-transcriptase polymerase chain reaction (RT-PCR) for SIV *gag* sequences [59] on weeks 0, 1, 2, 4, 8, and at monthly intervals thereafter. Assay sensitivity was determined to be 50 viral RNA copies/ml. Additionally, primers/probes and conditions according to Lifson were used [60].

### PCR and sequencing analysis

Chromosomal DNA from 10^6^ PBMC was extracted from animal ROm-8 at peak viremia (2 weeks after receiving infected blood from the penultimate RM) using a DNAzol genomic DNA isolation kit (Molecular Research Center Inc., Cincinnati, OH). To analyze the molecular evolution of SHIV-1157ipEL-p *env* during in vivo passaging, two different primers were synthesized to amplify the *env* gene (approximately 2.2 kb) of SHIV-1157ipEL-p that was isolated from monkey ROm-8 at 2 weeks post-inoculation. The following pair of primers was used: forward (5′ -AGTCTATTATGGGGTACCTGTATGGAAAGAAGCA-3′) and reverse (5′-TCCCAGATAAGTGCCAAGGATCCGTTCACTAATC -3′); PCR was carried out under end-point dilution conditions. The amplified fragment was cloned into the *KpnI* and *BamHI* sites of a pcDNA6/myc-His B vector for sequencing. DNA sequencing was performed for five randomly selected clones encoding an e*nv* gene.

### Generation of a large-scale SHIV-1157ipEL-p stock

A large-scale stock of the uncloned biological virus isolate was prepared by cocultivation of SHIV-1157ipEL-p-infected PBMC derived from monkey ROm-8 two weeks post-inoculation (the time of peak viremia) with naïve RM PBMC. The latter had been stimulated with concanavalin A and were cultured in the presence of human interleukin-2 (IL-2) (20 U/ml) and TNF-α (10 ng/ml). The resulting stock has a p27 concentration of 50 ng/ml and 1.5×10^5^ 50% tissue culture infectious doses (TCID_50_) per ml, as titrated in TZM-bl cells [14].

### Neutralization assays

The neutralization sensitivity of SHIV-1157ipEL-p was determined using the TZM-bl reporter cell line-based neutralization assays as described previously [7], [15]. TZM-bl cells (also called JC53-bl [14] cells (ARRRP) stably express CD4 and CCR5 as well as luciferase and β-galactosidase under the control of the HIV-1 LTR. In brief, virus was added to cells in the presence of DEAE dextran (40 µg/ml), washed 1x on day 1 and measured in a luminometer (Perkin Elmer, Waltham, MA) on day 2 after adding luciferase substrate (Bright-Glo, Promega, Madison, WI).

Serial dilutions of either RM sera or mAbs were prepared in triplicate in 96-well plates; virus was added (50–200 TCID_50_) and incubated for 1 h at 37°C before adding to the TZM-bl plates. For assays that employed immune sera, neutralization titers were calculated based on virus production in wells containing sera pooled from four naive RM as negative controls. The concentration of serum giving 50% neutralization of virus production (IC_50_) was calculated using the level of virus production in control wells that contained the same dilution of pooled naive sera. For mAb titers, IC_50_ was calculated based on control wells containing virus and cells only (i.e., no serum).

### Sequence and structural analysis

N-linked glycosylation sites were tracked using the LANL N-Glycosite program (http://www.hiv.lanl.gov/content/sequence/GLYCOSITE/glycosite.html). Potential N-linked glycosylation (PNG) sites were mapped on a model structure of gp120. Protein modeling and energy calculations were performed using Discovery Studio (Accelrys Software, Inc.) based on a sequence alignment with the core structure corresponding to the X-ray structure of YU2 [20] (pdb code 1RZK). V1, V2, and V3 loops were added to the core structure as previously described [37] and refined by the loop refinement program. Energies were calculated using CHARMM (Chemistry at Harvard Macromolecular Mechanics). We introduced solvent factors using the implicit of distance-dependent dielectrics model and performed energy minimization of Env loops (Steepest Descent following by Conjugate Gradient).

### Nucleotide sequence accession number

The complete nucleotide sequence of the *env* gene of SHIV-1157ipEL-p has been submitted to Genbank; an accession number is HM585377.

### Site-directed mutagenesis to create the mutant SHIV-1157ipEL-pΔ3N

To create the NNN deletion mutant, SHIV-1157ipEL-pΔ3N, from SHIV-1157ipEL-p, two different sets of primers were synthesized. Forward (5′ -TCTATTATGGGGTACCTGTATGGAAAGAAG-3′); reverse (5′-TAACACCACTTGATGAGAATTCTAGTGAGTATAGATTAAT-3′) and forward (5′-ATTAATCTATACTCACTAGAATTCTCATCAAGTGGTGTTA-3′); reverse (5′-TAAGTGCCAAGGATCCGTTCACTAATCGAA-3′). The full-length SHIV-1157ipEL-p *env*, cloned into the vector pcDNA6/myc-His B (Invitrogen, Carlsbad, CA), was used as template to create mutant SHIV-1157ipEL-pΔ3N *env* using overlapping PCR. The amplified fragment was cloned into the *KpnI (*K*)* and *BamHI (*B*)* sites of a pcDNA6/myc-His B vector and confirmed by sequencing. An infectious molecular clone of SHIV-1157ipEL-pΔ3N was generated using a similar strategy as that described above for SHIV-1157ipEL.

### Intrarectal inoculation of SHIV-1157ipEL-p and isolation of mucosal lymphocytes

Six animals received repeated weekly low-dose intrarectal (i.r.) inoculations (up to a maximum of 5): 5,000 TCID_50_ (two RM), 8,000 TCID_50_ (two RM) and 10,000 TCID_50_ (two RM). Monkeys that remained either aviremic or had only transient, low-level viremia (<10^4^ copies/ml) at the 2-week time point after the 5^th^ low-dose virus exposure were given a single high-dose i.r. challenge (1.5×10^5^ TCID_50_). Blood was collected at 0, 1, 2, 4, 8, and 12 weeks post-inoculation to determine viral RNA loads and T-cell subsets. PBMC were isolated by standard procedures; lymphocytes from rectal biopsies were obtained by digestion with collagenase followed by a separation step using Percoll gradients as described previously [11]. For subset analyses of T cells, approximately 1×10^6^ PBMC or lymphocytes from rectal biopsies were surface-stained as described [11].

### Statistical analysis

Prism® (GraphPad Software, version 4, CA) was used to compare percentages and numbers of lymphocytes between groups of uninfected and SHIV-1157ipEL-p-infected animals using the Mann-Whitney test.
